# Diosgenin reduces phosphodiesterase 3B (PDE3B) through AMP-activated protein kinase/ mechanistic target of rapamycin (AMPK/mTOR) signaling pathway to ameliorate streptozotocin-induced pancreatic β-cell apoptosis and dysfunction

**DOI:** 10.1080/21655979.2021.2023996

**Published:** 2022-01-14

**Authors:** Lijie Ma, Chengfei Zhang, Lili Wu, Lingling Qin, Tonghua Liu

**Affiliations:** a School of Traditional Chinese Medicine, Beijing University of Chinese Medicine, Beijing, P.R. China; b School of Traditional Chinese Medicine, Ningxia Medical University, Yinchuan, P.R. China; c School of Life Sciences, Beijing University of Chinese Medicine, Beijing, P.R. China; d Key Laboratory of Tcm Health Cultivation of Beijing, Beijing University of Chinese Medicine, Beijing, P.R. China; e Technology Department, Beijing University of Chinese Medicine, Beijing, P.R. China

**Keywords:** Type 2 diabetes mellitus (T2DM), diosgenin, phosphodiesterase 3B (PDE3B), streptozotocin (STZ)

## Abstract

Diabetes mellitus is a metabolic disease caused by defective insulin secretion and/or insulin action. And insulin is the main hormone released by the pancreatic β-cells. Diosgenin (DG) is a phytochemical with pharmacological activity that increases insulin secretion in streptozotocin (STZ)-induced pancreatic β-cells of diabetic rats. In this paper, we investigated the effect and mechanism of DG on cell apoptosis and dysfunction in STZ-induced pancreatic β-cells. Cell viability was detected by CCK-8, apoptosis by flow cytometry, and apoptosis-related protein expression by Western blot. Western blot and RT-qPCR were performed to detect the expression of related genes. The results showed that in STZ-induced INS-1 cells, DG could improve cell viability, inhibit apoptosis, attenuate oxidative stress levels and increase insulin secretion. Notably, PDE3B was highly expressed in STZ-induced INS-1 cells, while DG could significantly inhibit PDE3B expression in a dose-dependent manner. More importantly, overexpression PDE3B remarkably reversed the effect of DG on STZ-induced INS-1 cells. It is thus clear that DG might inhibit STZ-treated pancreatic β-cell apoptosis and reduce dysfunction via downregulating PDE3B, which provided a more reliable theoretical basis for the treatment of diabetes mellitus with DG.

## Introduction

Diabetes is a metabolic disease featured by hyperglycemia due to defects in insulin secretion and/or insulin action [1]. The development of diabetes involves multiple pathogenic processes, including autoimmune destruction of pancreatic B cells and subsequent insulin deficiency, as well as abnormalities that lead to resistance to insulin action [1,2]. Based on the etiology and pathology, diabetes is classified as type 1 diabetes mellitus (T1DM), type 2 diabetes mellitus (T2DM), gestational diabetes mellitus (GDM) and other [2]. Of these, type 2 diabetes is a heterogeneous metabolic disorder, the most common type of diabetes, characterized by insulin deficiency and insulin resistance [3]. Notably, insulin is the primary hormone released by the pancreatic β-cells [4]. Therefore, it is essential to study pancreatic β-cell dysfunction for the treatment of diabetes mellitus.

Diosgenin (DG) is a phytochemical with multiple disease-fighting activities that is widely found in legumes such as fenugreek and yam [5]. Due to its anti-cancer, anti-thrombotic, anti-neurological, anti-aging and anti-inflammatory properties, DG is efficacious against a wide range of pathological conditions, including cancer, hyperlipidemia, cardiovascular disease, diabetes mellitus, oxidative stress and inflammation [6–9]. It has been shown that DG significantly reduced the level of the glycolytic enzyme glucokinase in a diabetic rat model and returned to normal after 30 days of treatment; furthermore, it was found that the number of β-cells and insulin granules in streptozotocin (STZ)-induced diabetic rats increased after 30 days of DG treatment; thus, DG has a significant effect on insulin secretion and β-cell regeneration in STZ-induced diabetic rats with potential effects [10]. However, the mechanism of action of DG in the treatment of diabetes mellitus has not been studied in depth.

Of concern is that phosphodiesterase 3B (PDE3B) belongs to one of the PDE isozymes, and it has been suggested that PDE3B may play a key role in pancreatic β-cells, thereby affecting insulin secretion [11]. For example, inhibition of PDE3B by amrinone remarkedly augmented insulinotropic action of physiological glucose in pancreatic β-cells of normal rats [12]. Apelin inhibits insulin secretion in pancreatic β-cells by activation of PI3-kinase- PDE3B [13]. Meanwhile, it has been found that DG could inhibit excessive proliferation, migration and inflammatory response of synovial fibroblasts by targeting and downregulating PDE3B [14].

Therefore, this study constructed a cellular model of the disease through the STZ induction of pancreatic β-cells INS-1 and investigated whether DG ameliorated apoptosis and dysfunction in the model cells by regulating PDE3B.

## Materials and methods

### Cell culture and transfection

Rat insulinoma cell line (INS-1 cells, also known as pancreatic beta-cells) was obtained from National Infrastructure of Cell Line Resource (China). INS-1 cells were cultured in RPMI 1640 medium containing 10 mM HEPES, 5 mM glucose, 50 μM 2-mercaptoethanol and 10% fetal bovine serum, and incubated at 37°C with 5% CO_2_. Overexpression PDE3B plasmid (Ov-PDE3B) and pcDNA3.1 empty vector (Ov-NC) were obtained from Shanghai Gene Pharma company and transfected to INS-1 cells by Lipofectamine™ 2000 transfection reagent (Thermo Fisher Scientific, Inc.) according to manufacturer’s instruction.

### Cell viability

Cell viability was tested using the Cell Counting Kit-8 (CCK-8) (Solarbio Biotechnology Co., Ltd.) assay. INS-1 cells were inoculated in 96-well plates and then simultaneously incubated for 24 h with various concentrations (0.1, 1 and 10 μM) of DG (dissolved in dimethyl sulfoxide and diluted in RPMI-1640) and/or 3 mM STZ (dissolved in citrate buffer, pH 4.5 and diluted in RPMI-1640) [3,15]. Subsequently, 10 μl of CCK-8 solution was added to each well and incubated continuously for 4 h. Absorbance values at 450 nm were determined using an enzyme marker (Thermo Fisher Scientific, Inc.). In addition, cell viability was assessed using a lactate dehydrogenase (LDH) activity assay kit (Solarbio Biotechnology Co., Ltd.). Cell samples were processed according to the instruction of the kit and the absorbance was measured to calculate the relative level of LDH.

### Apoptosis assays

According to the Invitrogen™ Dead Cell Apoptosis Kit with Annexin V FITC and propidium iodide (PI) (Thermo Fisher Scientific, Inc.) instruction manual, Annexin V FITC and PI staining solution was added into each group of cell suspensions. Flow cytometry was then used to detect cell apoptosis in each group. The Q1 area indicates necrotic cells, Q2 area indicates advanced apoptotic cells, and Q3 area indicates early apoptotic cells.

### Western blotting assay

Cells were disrupted by lysis buffer and protein content was determined using the BCA Protein Assay Kit. The quantified protein samples were then separated by SDS-PAGE and transferred to PVDF membranes, followed by sealing with 5% skim milk for 1 hour at room temperature. The PVDF membranes carrying the samples were incubated with primary antibodies overnight at 4°C, and then with secondary antibody for 2 h at room temperature. The signal was displayed with an enhanced chemiluminescence (ECL) kit (Thermo Fisher Scientific, Inc.). And, the protein expression levels were semi-quantified using Image-Pro Plus version 6.0 (Media Cybernetics, Inc.) software. The antibodies used in this study were purchased from Abcam and were used at the following concentrations: anti-Bcl-2 (1:1000), anti-Bax (1:1000), anti-cleaved caspase3 (1:100), anti-caspase3 (1:500), anti-PDE3B (1:2000), anti-p-AMPK (1:1000), anti-AMPK (1:1000), anti-p-mTOR (1:1000), anti-mTOR (1:10,000), anti-GAPDH (1:2500), and goat anti-rabbit IgG H&L (HRP) (1:2000).

### Reverse transcription-quantitative PCR (RT-qPCR)

Total RNA was extracted from transfected cells by using the Trizol reagent (Invitrogen, Thermo Fisher Scientific, Inc.). cDNA was subsequently synthesized at 42°C for 30 min with the PrimeScript^TM^ RT Reagent kit (Takara Bio, Inc.). The PCR system was set up according to the instructions of the BeyoFast^TM^ Probe qPCR Mix kit (Beyotime Biotechnology Co., Ltd.). After pre-denaturation and amplification of the template (initial denaturation at 95°C for 10 min; followed by 40 cycles of denaturation at 95°C for 15 s and annealing at 60°C for 1 min; final extension for 10 min at 72°C), the results were analyzed using the quantitative PCR. The following primers pairs were used: PDE3B forward 5’-GTGCCGCCGAAGAAAAAGTG-3’ and reverse 5’- CAACTGCCATAGTAACTGGCTG-3’; insulin-1 forward 5’- GAGGCCATCAAGCAGATCAC-3’ and reverse 5’-TCCATCTCTCTCGGTGCAGG-3’; insulin-2 forward 5’-GGCCTTTGCGTCAGATCACT-3’ and reverse 5’- TGTTGGTTCACAAAGGCTGC-3’; GAPDH forward 5’-GGAGCGAGATCCCTCCAAAAT-3’ and reverse 5’-GGCTGTTGTCATACTTCTCATGG3’. Relative expression levels were compared using the 2^−ΔΔCq^ method.

### Analysis of insulin secretion and oxidative stress levels

Cellular insulin secretion was detected using the mouse insulin ELISA kit (Solarbio Biotechnology Co., Ltd.). Briefly, the medium of treated cells was centrifuged at 1000 × g for 10 min at 4°C. Subsequently, the release of insulin from cells in each group was assayed according to the operator’s manual. Oxidative stress indicators (including the levels of MDA, SOD and GSH-Px) in INS-1 cells were analyzed using the commercially available kits (Nanjing Jiancheng Bioengineering Institute Co., Ltd) according to the operation manual.

### Statistical analysis

Data were expressed as mean ± SD, and one-way ANOVA and Tukey’s test were performed with GraphPad Prism 8.0 software (GraphPad software, Inc.) to analyze differences. P < 0.05 was considered to indicate a statistically significant difference.

## Results

### DG increases cell viability and inhibits apoptosis in STZ-induced INS-1 cells

**Figure 1.**
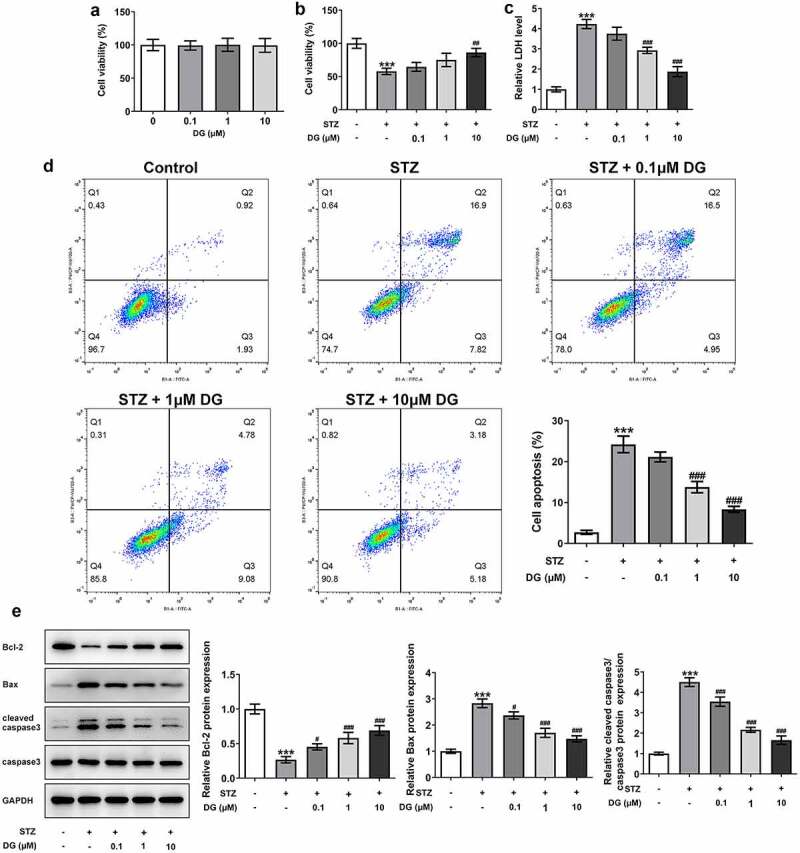
DG increases cell viability and inhibits apoptosis in STZ-induced INS-1 cells. (a) Results of cell viability with different concentrations of DG in INS-1 cells. (b) Results of cell viability with different concentrations of DG in STZ-induced INS-1 cells. (c) Results of LDH activity. (d) Apoptosis detection by flow cytometry. (e) Results of apoptosis-related protein expression, inculding Bcl-2, Bax, and cleaved caspase3/caspase3. ***P < 0.001 vs. control; ^#^P < 0.05, ^####^P < 0.01 and ^###^P < 0.001 vs. STZ.

Cell viability was detected by using CCK-8 and LDH release assay. The study showed that 0–10 μM DG treatment had no significant effect on INS-1 cell viability (Figure 1(a)). Compared with the control group, the cell viability reduced significantly after STZ induction, but improved significantly with increasing DG concentration from 0–10 μΜ (Figure 1(b)). LDH release assay showed that LDH levels in cells were enhanced upon STZ induction, and LDH release was significantly inhibited with increasing DG (Figure 1(c)). Apoptosis was analyzed by flow cytometry and Western blotting. Compared with the control group, apoptosis was increased on STZ induction, while apoptosis-related protein Bcl-2 expression was suppressed, and Bax and cleaved caspase3/caspase3 expression were upregulated (Figure 1(d,e)). In contrast, when DG concentration increased, apoptosis levels decreased in a dose-dependent manner with DG (Figure 1(d)). Meanwhile, apoptosis-related protein Bcl-2 expression was dose-dependently increased with DG, and Bax and cleaved caspase3/caspase3 were dose-dependently decreased (Figure 1(e)).

### DG improves insulin secretion and reduces oxidative stress levels in STZ-induced INS-1 cells

**Figure 2.**
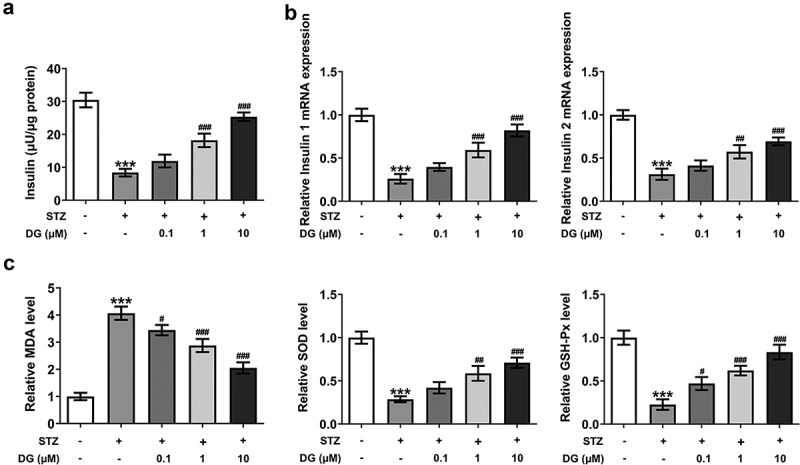
DG improves insulin secretion and reduces oxidative stress levels in STZ-induced INS-1 cells. (a) Results of insulin secretion levels. (b) Results of insulin gene level detection by RT-qPCR. (c) Results of oxidative stress indicators, including MDA, SOD and GSH-Px. ***P < 0.001 vs. control; ^#^P < 0.05, ^##^P < 0.01 and ^###^P < 0.001 vs. STZ.

The level of insulin secretion was detected by using the kit; the relative expression level of insulin 1 and insulin 2 was detected via RT-qPCR; and the levels of oxidative stress-related indicators were detected by the corresponding kits. The analytical results showed that STZ induction significantly inhibited insulin secretion, and the relative expression levels of insulin 1 and insulin 2 were significantly suppressed (Figure 2(a,b)). However, after an increase in DG, compared with STZ induction alone, insulin secretion levels were upregulated in a dose-dependent manner, as well as an increase in the relative expression levels of insulin 1 and insulin 2 (Figure 2(a,b)). In addition, the detection and analysis results for the levels of oxidative stress-related indicators showed that MDA levels were significantly increased in the STZ-induced group compared to the control group. MDA levels were significantly inhibited by the addition of DG in comparison to the STZ-induced group (Figure 2(c)). Conversely, the SOD expression level was suppressed by STZ induction and enhanced in a dose-dependent manner by the addition of DG (Figure 2(c)). Meanwhile, the trend of GSH-Px levels was similar to that of SOD (Figure 2(c)).

### DG inhibits PDE3B expression and affects AMPK/mTOR signaling pathway

**Figure 3.**
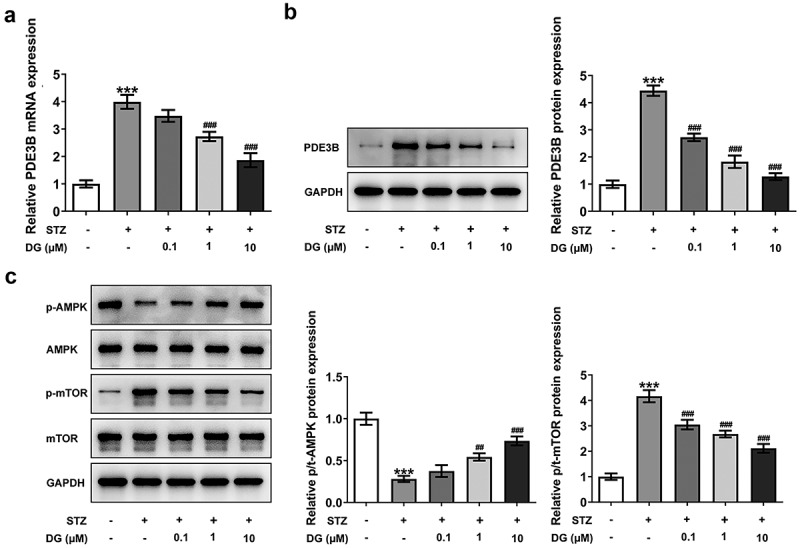
DG inhibits PDE3B expression and affects AMPK/mTOR signaling pathway. (a) The PDE3B mRNA expression in STZ-induced INS-1 cells. (b) The PDE3B protein expression in STZ-induced INS-1 cells. (c) The related-protein expression of AMPK/mTOR pathway. ***P < 0.001 vs. control; ^##^P < 0.01 and ^###^P < 0.001 vs. STZ.

PDE3B expression levels in STZ-induced INS-1 cells were analyzed by RT-qPCR and Western blotting. The study revealed that PDE3B mRNA and protein expression levels were significantly increased in STZ-induced INS-1 cells, while PDE3B expression was reduced upon increasing DG (Figure 3(a,b)). The results of AMPK/mTOR signaling pathway related protein assay demonstrated that DG could reverse the inhibition of p/t-AMPK and promotion of p/t-mTOR caused by STZ in a dose-dependent manner, thus suggesting that DG might regulate AMPK/mTOR signaling pathway (Figure 3(c)).

### DG increases cell viability and inhibits apoptosis in STZ-induced INS-1 cells via inhibiting PDE3B

**Figure 4.**
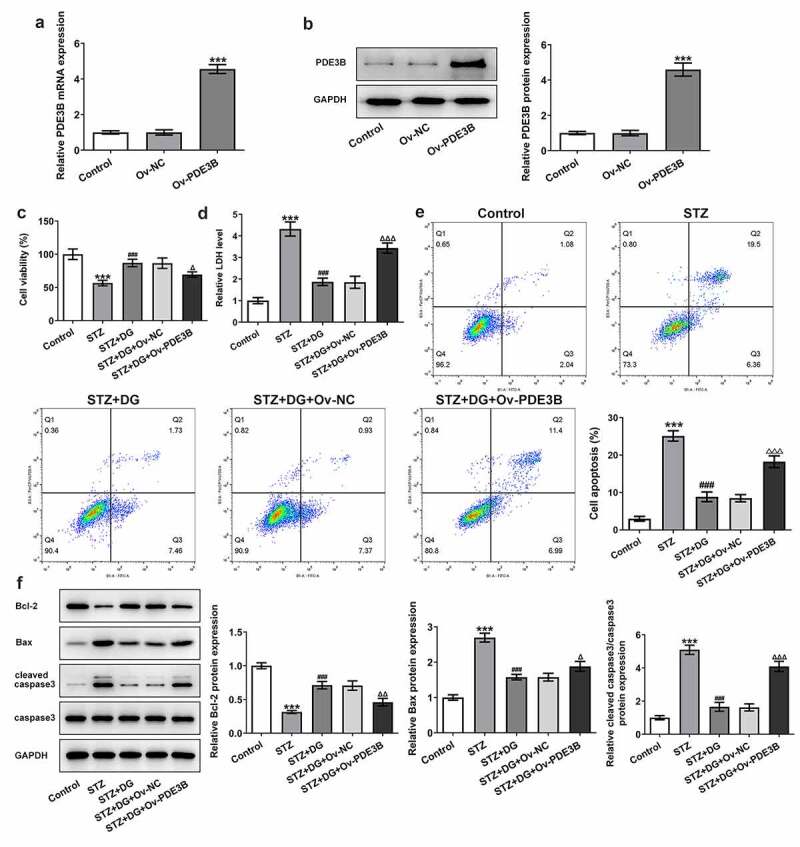
DG increases cell viability and inhibits apoptosis in STZ-induced INS-1 cells via inhibiting PDE3B. (a) The mRNA expression of PDE3B in INS-1 cells. (b) The protein expression of PDE3B in INS-1 cells. (c) Results of cell viability. (d) Results of LDH activity. (e) Apoptosis detection by flow cytometry. (f) Results of apoptosis-related protein expression, inculding Bcl-2, Bax, and cleaved caspase3/caspase3. ***P < 0.001 vs. Ov-NC or control. ^###^P < 0.001 vs. STZ. ^Δ^P<0.05, ^ΔΔ^P<0.01 and ^ΔΔΔ^P<0.001 vs. STZ+DG+Ov-NC.

To further demonstrate that DG exerted its effect by suppressing PDE3B, PDE3B overexpression cells were constructed from transfection and examined cell viability under the effect of DG. The mRNA and protein levels of PDE3B were significantly increased after transfection of Ov-PDE3B plasmids into INS-1 cells (Figure 4(a,b)). In addition, due to the largest difference in the effect of 10 μM DG in STZ-induced cells, subsequent experiments were carried out using 10 μM DG as the study concentration. The addition of DG was found to significantly increase cell viability compared to the STZ group; however, the effect of DG on cell viability was significantly reversed with PDE3B overexpression (Figure 4(c)). In contrast, STZ significantly promoted LDH release, which was inhibited by DG addition, while the inhibitory effect of DG was reversed in response to PDE3B overexpression (Figure 4(d)). In addition, for apoptosis studies, DG was observed to remarkably inhibit the apoptotic effect induced by STZ, but this inhibition was reversed in the PDE3B overexpression group (Figure 4(e)). The results of the apoptosis-related protein expression assay via Western blotting indicated that DG attenuated the effect of STZ induction for apoptosis-related protein, while the effect of DG was markedly reversed following PDE3B overexpression (Figure 4(f)).

### DG improves insulin secretion and reduces oxidative stress levels in STZ-induced INS-1 cells via inhibiting PDE3B

**Figure 5.**
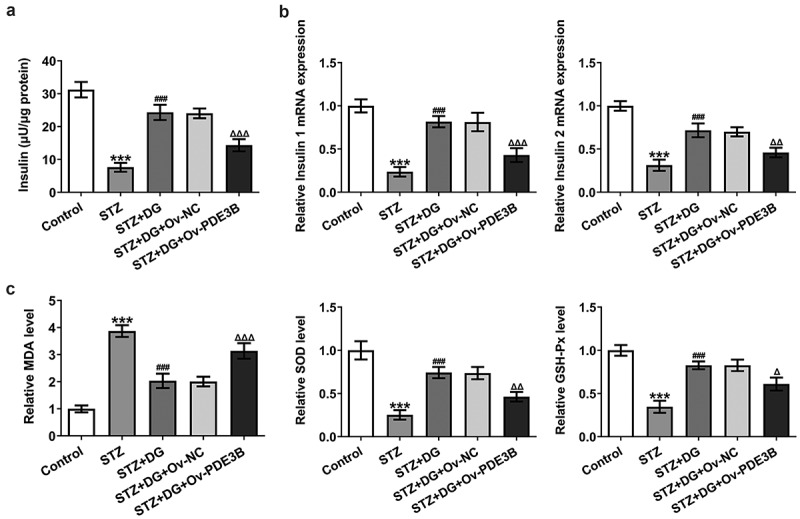
DG improves insulin secretion and reduces oxidative stress levels in STZ-induced INS-1 cells via inhibiting PDE3B. (a) Results of insulin secretion levels. (b) Results of insulin gene level detection by RT-qPCR. (c) Results of oxidative stress indicators, including MDA, SOD and GSH-Px. ***P < 0.001 vs. Ov-NC or control. ^###^P < 0.001 vs. STZ. ^Δ^P<0.05, ^ΔΔ^P<0.01 and ^ΔΔΔ^P<0.001 vs. STZ+DG+Ov-NC.

To demonstrate that DG targets PDE3B for effect, insulin secretion and oxidative stress-related analyses were again performed. The results suggested that DG could enhance insulin release in STZ-induced cells. But once PDE3B was overexpressed, the insulin-promoting effect of DG was reversed (Figure 5(a)). Moreover, the relative expression levels of insulin 1 and insulin 2 were similar to the trend of insulin secretion (Figure 5(b)). In the oxidative stress-related analysis, it was found that DG could markedly attenuate the significant differential variation in MDA, SOD and GSH-Px induced by STZ, and conversely, overexpression of PDE3B could significantly reverse these trends (Figure 5(c)).

## Discussion

DG is an important dietary steroidal saponin element extracted mainly from the seeds of fenugreek in the leguminous family [5,16]. Diabetes mellitus is a metabolic disease characterized as hyperglycemia caused by deficient insulin secretion or impaired insulin effect [9,17]. Crucially, pancreatic beta-cell injury was central to the pathogenesis of diabetes mellitus [18]. Therefore, in the present study, STZ-induced pancreatic β-cell INS-1 damage was used to mimic diabetes, and the effect of DG on STZ-induced pancreatic β-cells was investigated to further explore the mechanism of action of DG on diabetes treatment.

In a previous report, Kanchan et al. studied an animal model of STZ-induced diabetes in rats and found that DG significantly improved levels of oxidative stress in STZ-induced diabetic rats, leading to a decrease in lipid peroxidation and an increase in endogenous antioxidant levels in a dose-dependent manner [19]. Meanwhile, the investigators proposed that DG has the ability to modulate multiple molecular targets, especially oxidative stress and inflammation [20–22]. Furthermore, in a report on the combination of morroniside and DG in the treatment of high glucose-induced cardiomyocyte injury, it was demonstrated that DG had effects on increasing cell viability, inhibiting apoptosis and reducing reactive oxygen levels [23]. Similarly, in the present study, DG was found to not only increase STZ-induced pancreatic β-cell viability and inhibit apoptosis, but also attenuate oxidative stress levels. Of interest is that DG could increase insulin secretion in a dose-dependent manner, whereas the induction of STZ resulted in a decrease in insulin secretion. This is similar to the results of Kiss et al. who found that Fenugreek (a traditional herbal medicine containing DG) increased insulin secretion [24].

To further investigate the effect of DG on STZ-induced pancreatic β-cell dysfunction, PDE3B was introduced into the study as a possible target gene for DG action. Notably, it has been proved that increasing intracellular cAMP concentration promotes insulin secretion from pancreatic beta cells [11]. Furthermore, PDE3B is one of the enzymes that hydrolyze cAMP and cGMP and is often expressed in cells with important roles in the regulation of energy metabolism, including beta cells, hepatocytes, adipocytes and hypothalamus cells [25,26]. In addition, the inhibition of adipocyte lipolysis by insulin has been reported to be essential for the energy homeostasis of the body [27]. Disruption of this effect might lead to insulin resistance and T2DM. Importantly, the study found that the main target of the antilipolytic effect of insulin was PDE3B, whose phosphorylation by Akt led to an accelerated degradation of cAMP [28]. Excitingly, it was found and demonstrated in the present study that overexpression of PDE3B, in the presence of the same concentration of DG, reversed the effects of DG. For example, the protective effect of DG on cell damage was reversed with PDE3B overexpression compared to the STZ-induced pancreatic β-cell group treated with DG alone. And PDE3B overexpression was more prone to promote apoptosis, such a trend was consistent with the results reported by the current investigators that inhibitors of PDEs could prevent cell apoptosis and improve cell viability in STZ-induced pancreatic β cells [29,30]. More importantly, the present findings suggest that DG might serve as a PDE3B inhibitor to regulate oxidative stress and promote insulin secretion.

Moreover, the AMPK/mTOR signaling pathway has been reported to be present in T2DM by several individuals [31,32]. The research report suggests that PDE3B, an important regulator of cAMP signaling in cells, might inhibit AMPK activation [33]. In contrast, the attenuation of AMPK activation was reversed after PDE3B inhibitor treatment [34]. Meanwhile, in the present study, AMPK activation was attenuated after STZ induction, and inversely mTOR expression was increased. However, as the concentration of DG increased, AMPK activation was enhanced and mTOR expression was reduced.

## Conclusion

In brief, the study revealed that DG promoted STZ-induced pancreatic β-cell viability, inhibited apoptosis, enhanced insulin secretion and attenuated oxidative stress levels through suppressing PDE3B expression. And it is further proposed that DG reduced PDE3B improved STZ-treated pancreatic β-cell dysfunction via AMPK/mTOR signaling pathway. This might provide a more favorable theoretical basis for the treatment of DG in diabetes mellitus.

## Highlights

- Diosgenin inhibits PDE3B expression and affects AMPK/mTOR signaling pathway.
- Diosgenin increases cell viability and inhibits apoptosis in STZ-induced pancreatic β-cells via PDE3B.
- Diosgenin improves insulin secretion and reduces oxidative stress levels in STZ-induced pancreatic β-cells via PDE3B.
